# Meta-analysis of the increase in height in maxillary sinus elevations with osteotome

**DOI:** 10.4317/medoral.16921

**Published:** 2011-12-06

**Authors:** Rocío Antonaya-Mira, Cristina Barona-Dorado, Natalia Martínez-Rodríguez, Esther Cáceres-Madroño, José M. Martínez-González

**Affiliations:** 1 Resident in the Master’s in Oral and Dental Implant Surgery program at the University Hospital of Madrid; 2 Associate Professor of Oral Surgery in the School of Dentistry at the Complutense University of Madrid. Assistant Director of the Master’s in Oral and Dental Implant Surgery program at the University Hospital of Madrid; 3 Honorary Collaborating Professor at the Complutense University of Madrid; 4 Full Professor of Maxillofacial Surgery in the School of Dentistry at the Complutense University of Madrid. Head of the Department of Oral and Dental Implant Surgery at the University Hospital of Madrid

## Abstract

Objectives: To compare the different variations of sinus elevation techniques with osteotomes, to evaluate the increase in height achieved, and to quantify the osseointegration periods and the success rates for the implants placed. Study Design: A meta-analytic study with descriptive statistics was carried out on sinus elevations using osteotomes, analyzing a total of 11 articles published between the years 2003 and 2008. Results: Summers’ classic technique for performing sinus elevations with osteotomes differs from the current techniques being used with respect to the use of drills, the manner in which the sinus floor is fractured and how the sinus membrane is lifted, and especially on the type of graft used—the most current tendency being not to use a graft. The maximum gain in height is 4.62 mm, and the minimum gain in height is 2.07 mm, starting with a maximum residual bone height of 8.8 mm and a minimum of 4.1 mm. The osseointegration period is 4.9 months and the success rate is 95.5%. Conclusions: Performing sinus elevations with osteotomes is a predictable technique that enables achieving an increase in bone height and successful results, similar to those of other techniques used, in the placement of implants.

** Key words:**Osteotomes, maxillary sinus elevation, dental implants, osseointegration.

## Introduction

Today in dental implant surgery, we find ourselves with several challenges that we must resolve every day at the clinic. One of the most frequent challenges is atrophy of the posterior maxilla due to pneumatization factors (sinus growth and expansion), resorp-tion due to prolonged edentulism and biological aging (a decrease in osteoblast and osteoclast capacity, and a decrease in miner-alization and vascularization). Expansion of the maxillary sinus occurs in the inferior and lateral parts, and can even spread to-wards the lateral pyriform ridge of the nose, in the region of the canine eminence. As a result of all of these changes, there is a significant decrease in the height of the bone available in the posterior section of the upper arcade. Consequently, we often end up with a residual bone height of less than 10 mm between the alveolar ridge of the crest and the sinus floor.

In 1974, Tatum (1) developed a modified Caldwell Luc procedure for placing grafts in the maxillary sinus floor, which was pre-sented in 1977 at the Annual Meeting of the Alabama Implant Study Group, in Birmingham. This sinus elevation technique con-tinued to evolve until a way was found in which implants could be placed simultaneously.

In the early 1990s, Summers developed a technique called bone-added osteotome sinus floor elevation (BAOSFE), in which the particles of material displaced the sinus membrane apically and thus enabled achieving an adequate height for the placement of the implants. This technique was proposed for a residual bone height (RBH) of 5-7 mm (2).

At the Consensus Conference on Maxillary Sinus Elevation in 1996 (3), the members made the following recommendations which depend on the residual bone height (RBH):

• Category A (RBH ≥ 10 mm): classic implant procedure

• Category B (RBH ≥ 7-9 mm): osteotome technique with simultaneous placement of implants 

• Category C (RBH ≥ 4-6 mm): maxillary sinus elevation with lateral access and bone graft and immediate or deferred placement of implants

• Category D (RBH ≥ 1-3 mm): maxillary sinus elevation with lateral access and bone graft and deferred placement of implants.

## Material and Methods

We carried out a meta-analytic study based on the results obtained from a bibliographic search in PubMed on maxillary sinus elevations with osteotomes. The selection criteria were as follows:

- Publication between the years 2004 and 2010

- Articles with patients on whom the maxillary sinus elevations were performed using the osteotome technique with simultaneous or deferred placement of implants, with no restriction as to the number of cases.

Articles which only explained the maxillary sinus elevation technique using osteotomes and which did not show results in patients, meta-analytic studies or reviews, as well as articles in which the technique was carried out on cadavers, were excluded. The eleven articles selected were used for making a descriptive statistical analysis of the following variables:

• Number and sex of the patients

• Implants: number, length, thickness and treatment of the surface

• Surgical technique: analyzing the procedure for using drills and osteotomes, the fracture of the maxillary sinus floor, the decision to use or not to use bone graft and the type of bone graft used, where applicable, the bone height and the height achieved after the elevation. With regard to the complications, we analyzed the perforation of the membrane and therapeutic approach in such a case, in addition to the occurrence of benign paroxysmal positional vertigo (BPPV).

• Period of osseointegration

• Implant survival rate

## Results

We compiled a total of 1,219 patients, consisting of 287 men and 386 women, whereas the sex was not specified for the remaining 546 patients. A total of 2,063 implants were placed in the patients. Given that it was not possible to obtain all of the parameters outlined in the descriptive statistical analysis in each one of the articles, we analyzed a variable sample according to each parameter to be studied ( Table 1).

**Table 1:**
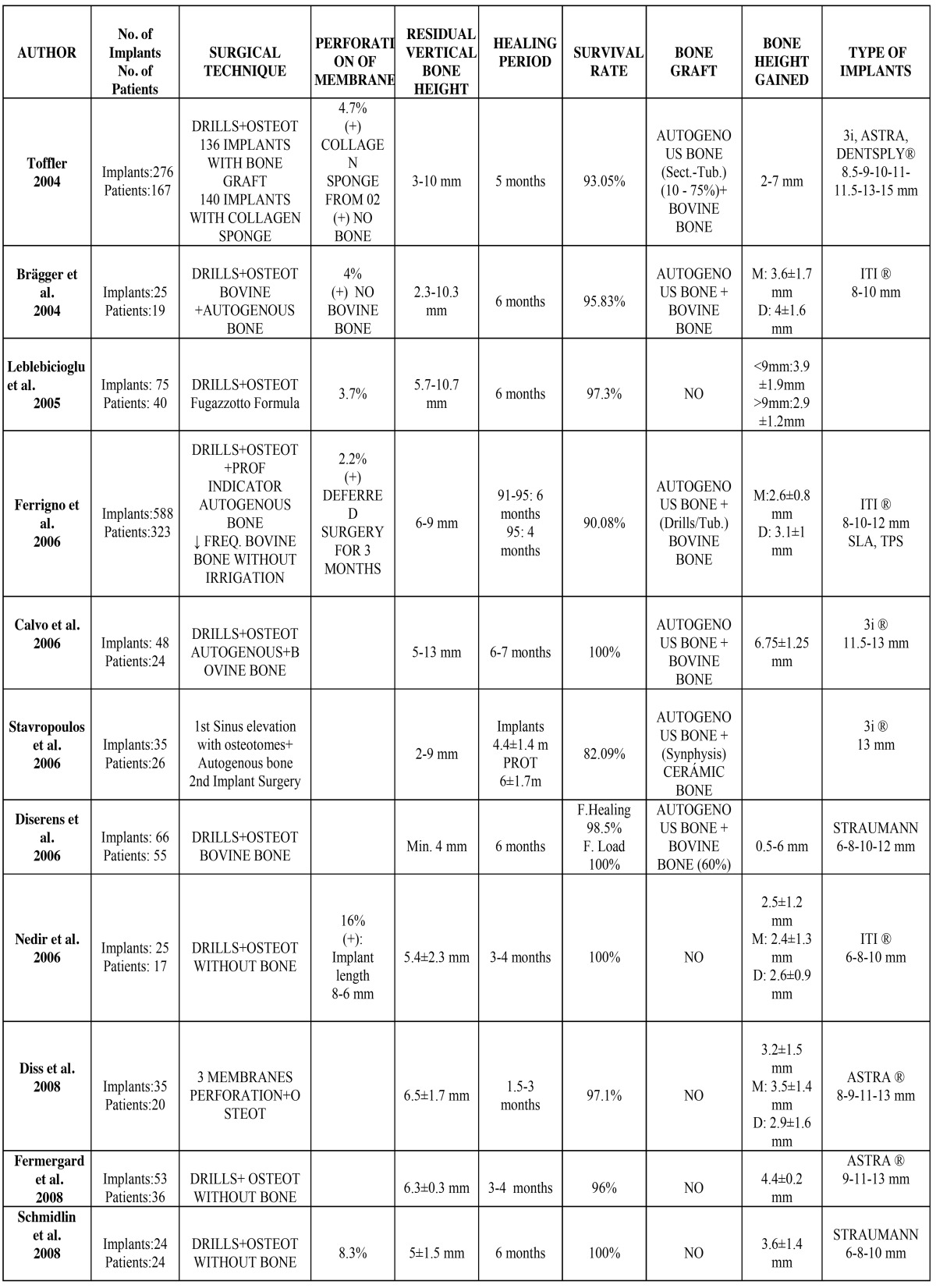
Detail of data from the articles selected.

**Figure 1:**
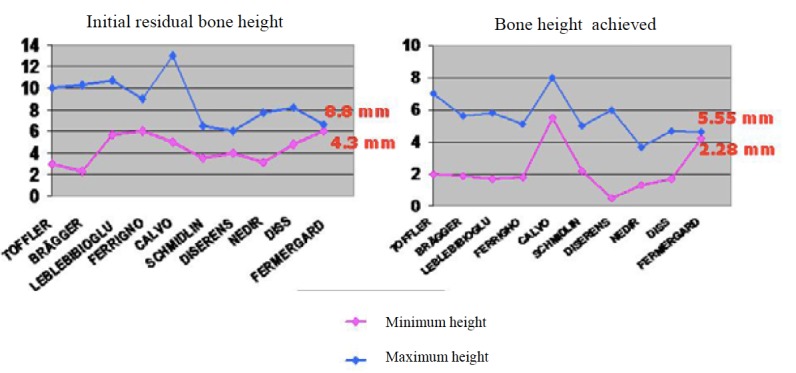
Comparison of the initial residual vertical bone height in the different studies and the gain in height achieved after sinus elevation with osteotomes.

The surgical technique was studied on a sample of 1,251 implants. In the majority of the literature, a combination of twist drills and used osteotomes up to 0.5-1.5 mm from the maxillary sinus floor were used. In 43% of the implants placed, the drilling was performed without any type of irrigation. In 2.5% of the implants, no drilling was used for the ostectomy. The fracture of the maxillary sinus floor was performed with osteotomes. Bone grafts were used in 83.1% of the implants placed, whereas in the remaining implants placed, the maxillary sinus elevation was carried out using osteotomes without any type of graft. In the implants placed using bone graft, it served as a cushioning for fracturing the maxillary sinus floor, reducing the risk of perforation of the sinus membrane. As for the type of bone graft used, bovine bone mixed with autogenous bone was used in 96.54% of the implants, whereas ceramic bone along with autogenous bone was used in 3.37% of the implants. In 0.1% of the cases, Demineralized Freeze-Dried Bone Allograft (DFDBA) was used. The maxillary sinus floor was fractured using osteotomes, adding bone graft in those cases where it was used, and using a surgical hammer to tap it lightly. In 11.11% of the implants analyzed, a collagen sponge was added in addition to the osteotomes in order to soften the fracture of the sinus floor. The elevation of the sinus membrane was one of the most delicate parts of the technique and it was performed using osteotomes, with the bone graft itself, if used, or with the depth indicators. The average initial residual bone height was a minimum of 4.3 mm and a maximum of 8.8 mm. The average bone height achieved was a maximum of 5.55 mm and a minimum of 2.28 mm (Fig. 1).

With regard to the complications associated with this technique, we have described two complications in our analysis: the perforation of the Schneider membrane during elevation and the subsequent onset of Benign Paroxysmal Positional Vertigo (BPPV). The perforation of the sinus membrane can go undetected in some cases, given that there is not a direct view of the perforation. This complication was analyzed on a sample of 1,013 dental implants and was observed to occur in an average of 6.5% of the cases. In the cases where perforation of the membrane was positive, no additional means were used in 9.7% of the cases; short implants (6.8 mm) were used in 2.5% of the cases; the surgery was deferred for 3 months in 58% of the implants; collagen sponges were used in order to cover the perforation in 13.82% of the implants; and no type of bone graft was used in 16.28% of the implants.

The second complication was the onset of BPPV after performing the maxillary sinus elevation with osteotomes. A sample of 813 implants was analyzed, in which the frequency of occurrence was 1.25%. A total of 2,063 implants were placed. When analyzing the length of the implants, the size was not specified for 46.2% of the sample. Taking a sample of 1,110 implants, the distribution in terms of the length of the implant was as follows: 6 mm in 0.27% of the sample; 8-9 mm in 15.94% of the sample; 9-10 mm in 39.63% of the sample; 11-12 mm in 31.08% of the sample; and more than 12 mm in 13.06% of the sample.

The average osseointegration period was calculated for a sample of 1,250 implants, obtaining an average of 4.8 months. The waiting periods varied greatly and there was no consensus among the different authors. The shortest osseointegration periods, ranging from 1.5 to 3 months, were reported for 2.8% of the implants. In 6.24% of the cases, the waiting period was 3-4 months. The longest osseointegration period was 4 months, which was the reported for 36.56% of the implants. Exceeding these osseoin-tegration times, a period of 5 months was reported in 22.08% of the cases, and a period of 6 months in 25.68% of the cases. For 6.64% of the implants, the waiting period ranged from 6 to 7 months.

**Figure 2:**
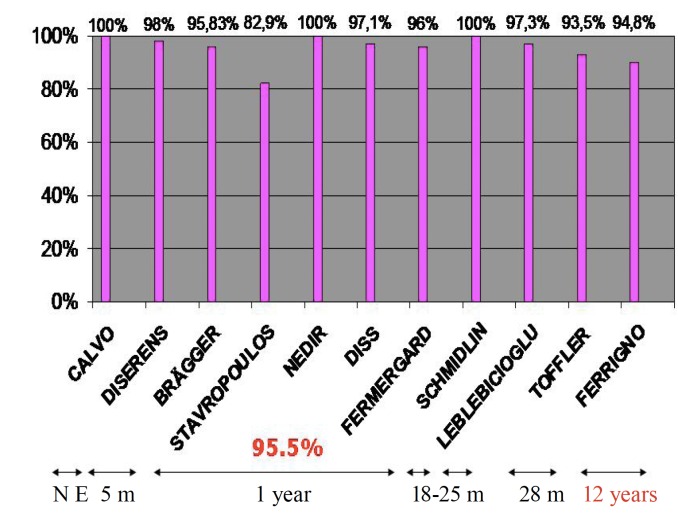
Survival rate of the studies, along with the different follow-up periods.

The survival rate for implants placed using the maxillary sinus elevation technique with osteotomes was calculated on a sample of 1,250 implants. Given that the follow-up time varied in the studies analyzed, the survival rates that were obtained also varied depending on the length of the follow-up time: 98% at 6 months; 94.1% at one year; 100% at 18 months; 97.3% at 25 months; 93.5% at 28 months; and 90.80% at 12 years (Fig. 2). The success rate at 12 years was only calculated in one study, obtaining a 90.8% success rate. The criteria used for calculating the survival rate in the studies were based on the implants that were func-tional and did not have any type of associated symptoms.

## Discussion

In accordance with the objectives outlined in our meta-analytic study, we analyzed the variations in the surgical technique used for performing maxillary sinus elevations with osteotomes.

Toffler (4) used drills and osteotomes up to 0.5-1.2 mm from the floor of the maxillary sinus. The procedure they used for frac-turing the sinus floor evolved during the course of their study. Initially, it was performed by filling the alveolus with bone graft and pushing it apically using an osteotome and a hammer. They later switched to the technique used by Cavicchia et al. (5), in which the maxillary sinus floor is perforated with the last osteotome. The integrity of the membrane was checked and a collagen sponge was placed apically, to which the bone graft was added. Leblebicioglu et al. (6) used the smallest diameter osteotome to fracture the sinus floor (7). Ferrigno et al. (8) used ostectomy drills without any type of irrigation, given that this enabled collecting the chips of autogenous bone from the drills in order to then use them to fill the bone graft. Stavropoulos et al. (9) used only osteotomes and a surgical hammer for preparing the ostectomy, without using any type of drill. They also performed the sinus elevation with osteotomes in the first surgery; and after a waiting period of 4-6 months, they performed a second surgery in order to place the implants.

With regard to the fracture of the maxillary sinus floor, there is a consensus among the authors studied, who perform it with os-teotomes and a surgical hammer, absorbing the tapping with the bone graft in those cases where it is used.

On analyzing the type of bone graft used, we observed how it was used in 83.1% of the implants, and we found that in 96.54% of these cases autogenous bone was mixed with bovine bone. Toffler (4) made the mixture in a proportion of 75/10% respectively, and they obtained it from the tuberosity or from the area behind the mandible. Brägger et al. (10) mixed the bovine bone with chips of autogenous bone obtained from the drilling, similar to that of Ferrigno et al. (8), who performed an ostectomy without irrigation; and Calvo-Guirado et al. (11), Diserens et al. (12) and Stavropoulos et al. (9) used a bone graft formed by the mixture of ceramic bone and autogenous bone derived from the mandibular symphysis. The current tendency is to perform maxillary sinus elevations with osteotomes, without using any type of bone graft (6,13-16).

The gain in height was analyzed on a sample of 1,215 implants. The initial average of the residual bone was a minimum of 4.3 mm and a maximum of 8.8 mm. It must be noted here that there are authors who performed sinus elevations with osteotomes when the minimum residual height was less than 4 mm (4,10,12,13,16). The bone height achieved varied greatly among the different authors. The minimum average increase in height was 2.28 mm, and the maximum was 5.55 mm. However, some authors achieved a maximum increase in height of more than 6 mm (4,11,12).

The complications analyzed were perforation of the sinus membrane and BPPV. Perforation of the sinus membrane was analyzed on a sample of 1,013 implants, and occurred in 6.5% of the cases. When this was positive, authors such as Leblebicioglu et al. (6) and Schmidlin et al. (16) did not use any additional means. On the other hand, Ferrigno et al. (8) preferred to defer the surgery by 3 months. Nedir et al. (13) placed short implants measuring 6-8 mm in the cases where the Schneider membrane had been perforated. Brägger et al. (10) avoided the use of bone graft in such situations. In the first years of his study, Toffler (4) placed a collagen sponge in the perforation and then inserted the bone graft and the implant. In recent years, however, similar to Brägger et al. (10), he did not use any type of bone graft in cases where the Schneider membrane had been perforated.

The onset of BPPV was analyzed on a sample of 468 patients and was found to occur in 2.13% of the cases. Its associated symptoms of vertigo accompanied by nystagmus have a varied etiology, which could be idiopathic or due to traffic accidents, ENT surgery, head injuries, surgeries with very long times in bed, vascular infections or disorders (17-20).

The osseointegration period was analyzed on a sample of 1,250 implants, obtaining an average waiting period of 4.8 months for osseointegration. There are also studies that exceed this average, such as the studies by Toffler (4), with a waiting period of 5 months; those of Brägger et al. (10), Diserens et al. (12), Schmidlin et al. (16), and Leblebicioglu et al. (6), with osseointegration periods of 6 months. In the first years of follow-up, Ferrigno et al. (8) reported waiting periods similar to those previously men-tioned; although in recent years, the waiting period for osseointegration decreased to 4 months, which is less than the average for this meta-analysis. Calvo-Guirado et al. (11) also reported a longer osseointegration period than the average for our study: be-tween 6-7 months. These findings are similar to those of Stavropoulos et al. (9), who reported the longest osseointegration period: a full 7 months. Those who obtained results below our average include the studies by Nedir et al. (13) and Fermergard et al. (15), with waiting periods of 3-4 months. The study with the shortest osseointegration period is that of Diss et al. (14), who reported 1.5 to 3 months. This data is due to the use of PRF membranes (Platelet-rich fibrin).

The survival rate of the implants was evaluated for the combination of all of the cases, although we have specified the follow-up time for each study, given that these varied among the different authors. With a follow-up time of one year, we obtained an aver-age success rate of 94.1% (10,9,13,14,15). The majority of the survival rate values are above 90%. Of particular note are the studies by Schmidlin et al. (16), with a follow-up period of 18 months and a survival rate of 100%; by Leblebibioglu et al. (6), with a follow-up period of 25 months and a survival rate of 97.03%; and Toffler (4), with a follow-up period of 28 months and a survival rate of 93.05%. The study with the longest follow-up period, 12 years, was by Ferrigno et al. (8), with a survival rate of 90.08%.
